# Developing Grit, Motivation, and Resilience: To Give Up on Giving In

**DOI:** 10.3390/pharmacy9020109

**Published:** 2021-06-09

**Authors:** Karen M. Whitfield, Kyle John Wilby

**Affiliations:** 1 School of Pharmacy, University of Queensland, Brisbane, QLD 4072, Australia; kwhitfield@pharmacy.uq.edu.au; 2 Faculty of Health, College of Pharmacy, Dalhousie University, Halifax, NS B3H 4R2, Canada

**Keywords:** resilience, motivation, burnout

## Abstract

Developing grit, motivation, and resilience within the pharmacy workforce has become a topic of increasing interest, heightened by the recent COVID-19 pandemic. Even prior to the global pandemic, the health care field has been associated with a rapidly changing, challenging, and pressured work environment that can often lead to stress and burnout. Developing resilience in health care workers has been identified as a strategy to combat burnout by improving their ability to thrive in stressful situations, thus enhancing physical and mental well-being. In this commentary, we consider the use of a resilience framework that encompasses the overlapping attributes of emotional balance and physical and mental strength to develop resilience. The importance of finding purpose and meaning is also explored within the framework, as well as the association between grit, motivation, autonomy, mastery, and connection. Practical strategies and reflections are outlined to challenge, inspire, and motivate the development of grit and resilience, in order to combat the challenges faced by pharmacists in a constantly changing health care system.

## 1. Introduction

If there was ever a year to look at the reasons why grit, motivation, and resilience are essential attributes for the pharmacy profession, 2020 was it. The COVID-19 pandemic had a global impact, creating unprecedented levels of challenge. Health care systems around the world experienced disaster management as never before, and the speed with which changes had to be made were overwhelming. The pharmacy profession played a major role in the fight against the pandemic, and many were involved in the management of medication shortages, development of treatment protocols, involvement in clinical trials, and implementation of antimicrobial stewardship [1,2,3]. Pharmacists played an essential role in continuing to deliver direct care and the provision of medications to patients. In particular, community pharmacists, often the most accessible health care practitioners, were placed under immense strain, as they experienced extreme workloads in order to enable the delivery of care to patients [4].

Prior to the COVID-19 pandemic, one author (K.M.W.) ran several international workshops on resilience and asked participants (predominately from the pharmacy profession) to identify the factors that they felt depleted or lowered their resilience. The similarity of factors outlined by different participants attending different workshops in different areas of the world was remarkable. Common factors included negativity often experienced in the workplace, managing expectations (self and managerial), experiencing knock backs, fear of failure, and rejection. Some examples of rejection were said to be rejection of a manuscript, unsuccessful career progression, or poor performance on a task or assignment. At the same time, there was large diversity in how people reported responding to challenges. Some people withdrew, stopped trying after rejection, and lowered expectations, while others grew stronger and developed new skills and rose to the challenge. It is interesting to draw comparisons with the concept of learned helplessness, which is a phenomenon observed in humans when they have experienced discomfort or suffering without a means of escape, and they eventually start to believe that they have no control over what happens and act as if they are helpless [5]. What causes people to respond differently, especially when the factors known to deplete resilience are so similar? One answer could lie within each individual’s own skill set of grit, motivation, and resilience. Perhaps those choosing to withdraw and lower their expectations are inherently lower on grit, motivation, and resilience than those who rise to the challenge. If so, how can one develop these qualities and skills to help improve their responses to the challenges encountered?

The purpose of this paper is as follows:

1. Explore the relationship between grit, motivation, and resilience.
2. Introduce a resilience framework, comprising four overlapping components of purpose, physical endurance, mental toughness, and emotional balance to assist in resilience development.
3. Encourage the reader to take a deeper dive into each of these components by providing a short introduction, a case study, and a challenge.

Grit is defined as a passion and perseverance for long-term goals that drives individuals to work through challenges, including failure and adversity, over a sustained period of time [6]. Related to this, motivation has been defined as the driving force behind which people strive to initiate and achieve their goals and to fulfil a need or uphold a value [7]. Both of these attributes play a significant role in the development of resilience. The American Psychological Association [8] defines individual resilience as the process of adapting well in the face of adversity, trauma, tragedy, or threats. It also includes coping with significant stress caused by problematic and toxic personnel or workplace relationships, serious health problems, and work or financial stressors. Resilience involves the capacity to bounce back from challenging situations but, it also provides the opportunity for personal growth. Resilience is not necessarily a personal trait, but involves behaviors, thoughts, and actions that an individual can learn and develop over time. The ultimate aim is to enhance overall well-being, which is defined by the WHO as a “state of complete physical mental and social well-being and not merely the absence of disease or infirmity”. In Kathryn McEwen’s book *Building Resilience at Work*, she describes a framework for building individual resilience that comprises four overlapping components of purpose, physical endurance, mental toughness, and emotional balance [9].

In the following section, the individual components of the framework will be discussed and we will highlight the importance of grit and motivation in the development of resilience. Each section will include a short case scenario and some reflections, together with a challenge-related exercise.

## 2. Purpose

Finding purpose in one’s life and work can lead to a high level of motivation and, in turn, may build resilience. For many, work needs to be more than a financial reward, people want to find meaning in their work. In other words, financial incentive may not be the key driver that prompts people to “bounce back” from challenging times. Instead, it may be their perceived purpose in the work that they do or the relationships that they form. Factors that have been associated with facilitating internal motivation include autonomy, mastery, and connection [10]. Autonomy is how an individual can direct their own work, while mastery describes the quest for competence, learning, and growth. Connection draws on interpersonal relations to create a sense of belonging. In a recent study investigating how motivation can stem clinician burnout, it was highlighted that at the center of mastery and autonomy lies purpose [11]. Articulating why people do the work they do is essential in order to understand purpose, and is thought to be directly related to resilience development.

### 2.1. Purpose Case Study (K.M.W.)

In 2013, I was attending an advanced leadership program, during which I was asked to write a personal purpose statement. At the time, I was working as a full time academic and this is what I wrote:

*“To assist people to achieve their full potential despite barriers and to build and nurture a highly effective sustainable research team incorporating both junior and senior people”*.

Today, nearly eight years later, working in a slightly different role that encompasses working as a clinician and an academic, having learnt much in those interim years about self-care, motivation, determination, and resiliency, my purpose statement reflects this:

*“To be an effective leader, to inspire others and make a difference whilst maintaining a balanced life”*.

Reflecting on this example, it can be seen that your purpose in life is not necessarily static, but will change with life experience, personal growth, and development. This example also demonstrates how autonomy and striving for competence play an important role in purpose. People who have a clear vision of what they are endeavoring and motivated to do are more likely to remain strong and work through the tough times. There is evidence to suggest that individuals who have a clear purpose have lower stress levels and a higher degree of resilience [12].

### 2.2. The Challenge: To Articulate a Personal Purpose Statement

Start by reflecting on the questions below, which will help identify what is important to you, your values, and the difference you want to achieve. Start to craft your personal purpose statement in 50 words or less. Details of how you will accomplish your purpose can be articulated in your goals.

- What bring happiness to my life?
- How do I want to make a difference?
- What legacy do I want to leave behind?
- What are my strengths?
- What are my top five core values?

A purpose statement will most likely change over time depending on what is happening in your life. Set aside time every year to review your purpose statement and make adjustments as required.

## 3. Physical Endurance

Participating in physical endurance has been associated with a more positive mindset as a result of endorphin release. Improving physical endurance has also been associated with lower levels of anxiety, depression, and stress. It is interesting that McEwen chose to use physical endurance and not simply physical fitness in the framework. It is useful when linking to grit and resilience development by adding the element of being able to press through a tough situation. Regular physical exercise has been associated with many advantages, including positive physiological and psychological benefits, protecting against the potential effects of stressful events, and protection against chronic diseases [13]. However, these messages remain insufficient to motivate us to get active and continue physical activity. Instead, the promotion of the mental health rewards from being active which can be immediate, may provide a better motivator. Benefits such as decreased stress, increased self-esteem, reduced levels of depression and anxiety, improved quality of sleep, and overall increased levels of energy may provide the incentive to not only start, but also maintain, a regular commitment to engage in physical activity and self-care [13]. Another approach to encouraging self-care, specifically related to health care workers, is to consider how this can enhance the care delivered to patients. A resilience training program to reduce burnout has been specifically developed for clinicians [14]. The authors saw the importance of self-care and designed several self-care and personal reflective questions to train and limit negative self-talk. One of the questions included “How can I take care of myself so that I can be of service to others?”. This may be another way to motivate and continue participation in more physical endurance, physical activity, and self-care.

### 3.1. Physical Endurance Case Study (K.J.W.)

Moving into an academic administrative position was challenging. New responsibilities to learn, increased workload, challenging interpersonal dynamics, and the never-ending emails and phone calls. I quickly learned that my “safe place” was either in the gym or out for a run. Listening to music and letting my mind drift away from work-related pressures for those 30 to 60 min was the self-care I needed to rejuvenate, do something good for myself, and approach work with a clearer frame of mind. The discipline to make a regular commitment to exercise was important, and those times when life was busy and the commitment waivered at times resulted in reduced efficiency and productivity.

Reflecting on this example, it can be seen that physical activity and self-care play important roles in combatting challenging situations by maintaining mental and physical wellness. Physical fitness helps develop resilience, as regular physical activity can produce positive physiologic and psychological benefits, which provide protection against the potential consequences of stressful events [13]. Participating in regular physical activity will look different to different people. Self-care should be looked at through a different lens and be seen as an investment that can lead to optimizing productivity, efficiency, and effectiveness.

### 3.2. The Physical Activity Challenge

The key to participating in physical fitness is to find exercise that is enjoyable and that can be built into a daily routine. Choose activities that you enjoy and that align with your lifestyle and abilities. One of the largest hurdles to overcome is making new habits or routines long-lasting. Use an evidence-based approach—start small and set small achievable and realistic goals. As these goals are met, self-confidence will increase, as well as momentum and motivation. Move to more challenging goals. In addition, using triggers is an excellent way to build a routine. These can be simple reminders, an alert or a specific time of day that acts as a cue, enabling your routine to become automatic.

Challenge yourself to commit to building physical activity into your everyday routine, and keep a diary to look back on your achievements and reflect on differences you see, for example, in energy levels, sleep, self-confidence, productivity, and efficiency.

## 4. Mental Toughness

Mental toughness has been studied extensively in the sports industry, and is associated with an individual’s ability to deal effectively with challenges and persist under pressure. Individuals with high levels of mental toughness often see challenges as opportunities for personal growth and learning [15]. Mental toughness is associated with greater well-being, lower perceived stress levels, and fewer depressive symptoms. Mental toughness, also described as grit, is gaining interest with other non-sport disciplines, including health professions and health professional educators [15]. The attributes associated with mental toughness, such as stamina, determination, persistence, endurance, achievement orientated, optimism, and excellence, can be seen as beneficial when working in health care organizations. Although there is some evidence that approximately 50% of variation in mental toughness is genetic [15], theories exist that developing mental toughness involves challenging yourself, learning from your failings, and believing that failure is not a permanent condition [16,17,18]. However, little is known about how to build it, so again we turn to the sporting world. In a study interviewing several elite athletes, some common underlying mechanisms associated with the development and sustainability of mental toughness were identified [18]. These included motivational factors, such as enjoyment and the need for mastery. Connection and a support network were also identified as important factors, as well as a desire to succeed. Importantly, the development of mental toughness was recognized as a trait that developed over time. These findings aligned with those of a study that interviewed a group of Paralympians about mental toughness, and found that their key characteristics included determination, optimism, self-belief, and autonomy [19]. Two themes emerge around mental toughness development, namely, (1) cognitive strategies, such as challenge, recovery from setbacks and failure, sustained commitment, mind-set, and acceptance, and (2) support resources, such as social support from others and reflection.

### 4.1. Mental Toughness Case Study (K.J.W.)

We recently had a manuscript rejected from a leading medical education journal that we collaboratively wrote with two other colleagues. As with many manuscript rejections, it can be tempting to withdraw without resubmission elsewhere based on the comments received, and move on to focus on the next manuscript. Alternatively, we could have lowered our expectations and submitted to a journal of a perceived lower quality, or simply felt the effort needed to change the manuscript was not worth the time we needed to invest. By relying on each other and our colleagues, we instead used the feedback provided to better the manuscript and talked through the challenging comments and our emotions together. We took it as an opportunity to improve our skills, specifically around how we chose to present the data. In the end, we submitted to another leading journal and were accepted.

Reflecting on this example, it can be seen that there is incredible value in collaboration and connection with others. Resilience is about asking for help and recognizing when your way is not working. It is about reaching out and connecting with others, because resilient people ask for help more often and have the highest number of positive relationships available when needed [20].

This case study also demonstrates the importance of failing in life and how failure is approached. The most resilient people have the trait of mental flexibility, to allow them to fail absorb, recover and review, change course, pick themselves up, and continue adapting to the environment [21]. Mental toughness and grit are the ability to persevere with something when things get tough. People with high levels of mental toughness can push beyond barriers towards success [22].

### 4.2. The Mental Toughness Challenge

Grit is defined as passion and perseverance for long-term goals that drives individuals to work through challenges, including failure and adversity, over a sustained period of time [22]. Challenge yourself to listen to the TED talk by Angela Duckworth titled *Grit—The Power of Passion and Perseverance* [23]. Start to reflect on situations in which you have experienced failure. Then start training yourself to think optimistically and find the positive in every situation, and for the next 30 days keep a journal to reflect and look back on.

## 5. Emotional Balance

Emotional balance is often described as the ability of the mind and body to maintain equilibrium and flexibility during times of adversity, stress, and change [9]. Developing emotional balance is key in developing resilience. Self-awareness, focus, and mindfulness are often said to be three interconnected skills that enable people to exercise emotional balance. To develop emotional balance, there is a need to become aware of emotions being experienced and to then be able to identify were they are coming from. At this stage, it is important to introduce mindfulness and emotional regulation to respond to these emotions with intention, rather than with an automatic or unconscious response. Commander Mark Divine, a retired Navy SEAL takes a similar, but slightly different, approach to developing emotional balance and discusses the concept of emotional resilience. In his book *Unbeatable Mind*, he describes strategies to assist maximizing peoples’ potential in order to enable leading a more balanced successful and happy life [24]. Part of his teaching is using a four-step emotional resilience framework:

1. Learn to recognize negative emotions as they start to arise and appear
2. Identify the root cause for the negative emotion
3. Intentionally transfer the negative root emotion to a positive counterpart
4. Engage the new positive emotion with self-talk, helping to divert attention to focus on something more positive.

Positive self-talk has been used extensively in the sports world, and places emphasis on attention control, increasing concentration, and reducing anxiety, with a view to challenging irrational beliefs [25]. Self-talk is becoming an increasing popular tool in health care. A workplace resilience program specifically designed to strengthen resilience in mental health nurses found that using positive self-talk strategies and challenging negative thoughts greatly assisted in reframing stressors, problem solving, and responding to challenging situations more effectively [26].

### 5.1. Case study Emotional Intelligence (K.M.W.)

Self-doubt, the fear of failure, and imposter syndrome are often highlighted in the workshops I run on resilience as factors that can deplete a person’s resilience. Recently, I had the opportunity to reflect on this when talking with a colleague about their reluctance to accept an opportunity to undertake some research in the workplace. On listening to their concerns, the beliefs of not being good enough and their fear of failing were evident.

Reflecting on this scenario, it can be seen that self-doubt occurs when there is a lack of confidence, when there is uncertainty and concerns about things not going to plan.

A degree of self-doubt is good, as it indicates an understanding that we are not always right and there is a need to improve and continually grow and develop. However, persistent self-doubt can allow people to become stuck and prevent them from achieving their full potential. The reasons for self-doubt can be numerous, including previous mistakes, childhood upbringing, and constantly comparing with others. The fear of failing is also common but, interestingly, a point raised by Elizabeth Gilbert in her book *Big Magic-Creative Living Beyond Fear* is about the fear of success and the fear of being able to replicate her success [27].

### 5.2. Emotional Balance Challenge

Familiarize yourself with Divine’s emotional resilience framework [24] and begin to start recognizing feelings of self-doubt. Prioritize time to reflect on your emotions, commit to regular journaling, and specifically write about actions taken to stop the negative consequences of self-doubt. These may include strategies such as positive self-talk, dealing with setbacks, or connecting with others. Make note of the strategies that work for you and focus on developing and refining them to best suit your own personal needs.

## 6. Summary

The COVID-19 pandemic has impacted everyone within the pharmacy profession, from undergraduate students, communities, hospitals, academics, and regulatory pharmacists. With significant changes to workforce and workflows, changed working environments, different ways to communicate, and the speed at which these changes had to be introduced, have impacted significantly on individuals, teams, and organizations. It has brought to the forefront the need to introduce resilience training into undergraduate curricula and continued professional development programs. Resilience is a complex concept and utilizing a framework may be helpful in identifying areas for individuals to work on in the journey towards developing resilience.

Having purpose provides perspective, motivation, and determination, which enables the ability to bounce back in challenging times. Developing physical fitness brings many health benefits and requires discipline in order to build it into a regular routine. Developing grit though mental toughness builds the capacity to be flexible and cope with the challenges of life and work. Self-awareness and the ability to recognize negative emotions, to then take action and turn into more positive thinking, is a skill that builds resilience. In addition, resilient people recognize the need to connect with others and work with likeminded positive people. When the traits of grit, motivation, and resilience are evident, then it becomes easier to give up on giving in.
